# Optimizing ceftolozane-tazobactam dosage in critically ill patients during continuous venovenous hemodiafiltration

**DOI:** 10.1186/s13054-019-2434-5

**Published:** 2019-04-26

**Authors:** Gerardo Aguilar, Rafael Ferriols, Sara Martínez-Castro, Carlos Ezquer, Ernesto Pastor, José A. Carbonell, Manuel Alós, David Navarro

**Affiliations:** 1 Critical Care Unit, Anesthesiology and Critical Care Department, Hospital Clínico Universitario de Valencia, Valencia, Spain; 2 INCLIVA Health Research Institute, Avenida de Menéndez y Pelayo, 4, 46010 Valencia, Spain; 3 Department of Pharmacy, Hospital Clínico Universitario de Valencia, Avenida Blasco Ibáñez, 17, 46010 Valencia, Spain; 4 Department of Microbiology, Hospital Clínico Universitario de Valencia, Avenida Blasco Ibáñez, 17, 46010 Valencia, Spain; 5 School of Medicine, University of Valencia, Avenida Blasco Ibáñez, 15, 46010 Valencia, Spain

Ceftolozane-tazobactam (C/T), the combination of a new cephalosporin with a classic β-lactamase inhibitor, is currently considered the most active betalactam antibiotic against *P. aeruginosa* [1]. Despite several case reports on C/T pharmacokinetics in critically ill patients during continuous renal replacement therapy (CRRT) [2–4], the optimal dose in this clinical scenario still remains unclear [5].

A 68-year-old patient was admitted to our ICU with septic shock (nosocomial peritonitis) and anuric acute renal failure. Broad-spectrum antimicrobial therapy, including C/T and continuous venovenous hemodiafiltration (CVVHD), was initiated, using a polysulphone hemofilter (Fresenius, Germany) with blood flow, dialysate fluid, and replacement fluid rates of 100 mL/min, 2000 mL/h, and 1000 mL/h. The patient received high C/T doses of C/T 2 g/1 g every 8 h (infused over 1 h) while receiving CVVHD, and became apyrexial 7 days after C/T treatment initiation, remaining fever-free for 14 days without any adverse effects related to this drug.

Pre-filter and post-filter blood and ultradiafiltrate samples were obtained during the 8-h dosing interval after the fourth dose. Drug concentrations were measured by high-performance liquid chromatography. Figure 1 and Table 1 show pre- and post-filter plasma concentrations. Pharmacokinetic parameters were calculated (Table 2). Extraction ratios were high for both ceftolozane and tazobactam (49.3% ± 1.8% and 40.5% ± 4.5%). Mean C/T concentrations in the ultrafiltrate were 40 mg/L and 13.5 mg/L, respectively.

**Fig. 1:**
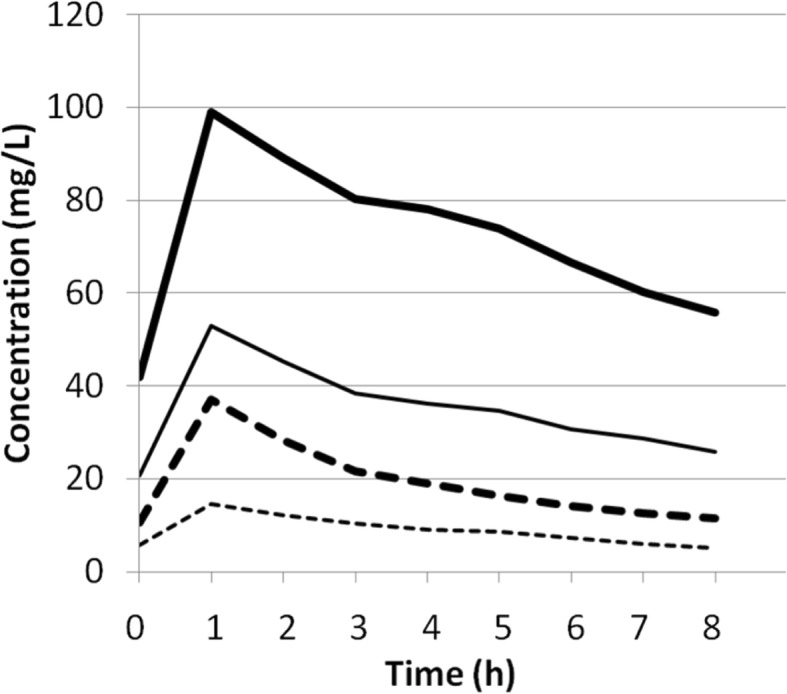
Simulated plasma concentrations versus time curves for ceftolozane and tazobactam. Pre-filter (thick line) and post-filter (fine line) ceftolozane plasma concentrations and pre-filter (thick dotted line) and post-filter (fine dotted line) tazobactam plasma concentrations. (The figure is original for this article)

**Table 1:** Concentrations of ceftolozane and tazobactam in pre-filter and post-filter plasma samples obtained after the fourth dose of 2 g/1 g ceftolozane-tazobactam administered as intravenous 1-h infusion

Sampling time	Ceftolozane (mg/L)		Tazobactam (mg/L)	
	Pre-filter	Post-filter	Pre-filter	Post-filter
0 h (pre dose)	41.9	20.7	10.6	5.8
1.5 h post dose	89.1	45.2	28.3	12.2
2 h post dose	80.3	38.4	21.6	10.3
2.5 h post dose	77.1	36.1	19.0	9.0
3 h post dose	73.8	34.7	16.3	8.2
5 h post dose	66.6	30.6	14.2	7.4
7 h post dose	60.2	28.7	12.7	6.0
8 h post dose	55.8	25.8	11.4	5.1

**Table 2:** Pharmacokinetic parameters of ceftolozane and tazobactam

Parameter	Ceftolozane		Tazobactam	
	Pre-filter	Post-filter	Pre-filter	Post-filter
Clearance (L/h)	2.1	5.4	6.4	17.4
Volume of distribution (L)	53.9	97.5	108.9	194.2
Half-life (h)	17.9	12.6	11.9	7.8
AUC (h mg/L)	960	373	157	57.6
Maximum concentration (mg/L)	99	53	37	14.5
Minimum concentration (mg/L)	55.9	25.8	11.4	5.1

*AUC* area under the concentration-time curve

We decided on a 3 g/iv dose every 8 h, taking into account two previous studies [3, 4] and a recent study which showed CRRT to be an independent predictor of clinical failure (OR 4.5, 95% CI 1.18–17.39, *p* = 0.02) when C/T is administered at 1.5 g every 8 h [5].

Ceftolozane and tazobactam are small molecules with low plasma protein binding rates, causing most to be removed during CRRT. Despite the considerable C/T clearance observed in our patients during CVVHD, however, ceftolozane plasma concentrations remained above the MIC, for MICs of up to 8 μg/mL, throughout the dosing interval, assuming 20% protein binding. Given that C/T exhibits linear, dose-proportional pharmacokinetics, a standard C/T dose of 1 g/0.5 g would be expected to maintain ceftolozane levels above the MIC during the entire dosing interval, although tazobactam concentrations could be insufficient, even taking higher pre-filter rather than lower post-filter levels as representative of therapeutic serum levels.

In conclusion, our data underscore that a dosage of 3 g every 8 h can be used safely to prevent the potential harm of underdosing ceftolozane/tazobactam during CRRT; larger studies are however needed to confirm our findings.

## Abbreviations

AUC: Area under the concentration-time curve
C/T: Ceftolozane-tazobactam
CRRT: Continuous renal replacement therapy
CVVHD: Continuous venovenous hemodiafiltration
HPLC: High-performance liquid chromatography
